# Characterizing the Genetic Basis for Nicotine Induced Cancer Development: A Transcriptome Sequencing Study

**DOI:** 10.1371/journal.pone.0067252

**Published:** 2013-06-18

**Authors:** Jasmin H. Bavarva, Hongseok Tae, Robert E. Settlage, Harold R. Garner

**Affiliations:** Virginia Bioinformatics Institute, Virginia Tech, Blacksburg, Virginia, United States of America; AC Camargo Cancer Hospital, Brazil

## Abstract

Nicotine is a known risk factor for cancer development and has been shown to alter gene expression in cells and tissue upon exposure. We used Illumina® Next Generation Sequencing (NGS) technology to gain unbiased biological insight into the transcriptome of normal epithelial cells (MCF-10A) to nicotine exposure. We generated expression data from 54,699 transcripts using triplicates of control and nicotine stressed cells. As a result, we identified 138 differentially expressed transcripts, including 39 uncharacterized genes. Additionally, 173 transcripts that are primarily associated with DNA replication, recombination, and repair showed evidence for alternative splicing. We discovered the greatest nicotine stress response by HPCAL4 (up-regulated by 4.71 fold) and NPAS3 (down-regulated by -2.73 fold); both are genes that have not been previously implicated in nicotine exposure but are linked to cancer. We also discovered significant down-regulation (-2.3 fold) and alternative splicing of NEAT1 (lncRNA) that may have an important, yet undiscovered regulatory role. Gene ontology analysis revealed nicotine exposure influenced genes involved in cellular and metabolic processes. This study reveals previously unknown consequences of nicotine stress on the transcriptome of normal breast epithelial cells and provides insight into the underlying biological influence of nicotine on normal cells, marking the foundation for future studies.

## Introduction

Worldwide, more than 1 million women are diagnosed with breast cancer every year and more than 410,000 die of the disease [1]. Large cohort studies performed in the United States and Japan indicate that the risk of breast cancer is associated with active and passive smoking [2], [3]. Studies have shown that 80–90% of inhaled nicotine is absorbed systemically during smoking, 1 mg from a single cigarette, resulting in plasma concentrations of about 15 ng/mL immediately after smoking [4]. In vivo studies demonstrate nicotine promotes the growth of solid tumors, suggesting that nicotine may contribute to cell proliferation, invasion, and angiogenesis [5]–[7]. Further, nicotine is shown to override DNA damage-induced cell-cycle G1/S restriction and thus promotes genetic instability [8]. Previous studies have shown that nicotine stimulation could alter gene expression in endothelial and neuroblastoma cells [9], [10]. A microarray based study linked nicotine stimulation with transcription factor NF-kB, but concluded that future analysis would be required since they evaluated only 4,132 genes and there was a strong possibility important genes were missed [9]. Another microarray study of neuroblastoma cells suggested that physiological and psychological effects of nicotine exposure may be due to the effects on gene expression, but they also had similar technical limitations [10]. Additionally, studies on nicotine lack consensus on nicotine dosage.

We hypothesize the missing link between nicotine stress and cancer will be found by using an unbiased sequencing approach rather than a targeted array based approach. Next-generation sequencing (NGS) techniques, in contrast with cDNA microarrays used previously, enables systematic examination of known, uncharacterized transcript expression over a wide dynamic range, and alternative and novel splicing events without any technological and/or biological bias. This all-inclusive approach may provide better clues to complex pathways, understanding of uncharacterized transcripts and provide missing information on gene regulation under nicotine stress that were previously not possible with microarrays. Moreover, we selected a LD_50_ dose because it is standardized and is established as an accurate means of measuring the effects [11]. Here, we describe the findings from this systematic analysis and discovered previously unknown associations of uncharacterized transcripts.

## Materials and Methods

### Reagents, chemicals and cell culture

Nicotine was purchased from Sigma (St. Louis, MO, USA). MCF-10A, normal breast epithelial cell-line, was obtained from American Type Culture Collection (ATCC, Manassas, VA). MCF-10A cells were cultured in DMEM/F12 medium (Invitrogen, Carlsbad, CA), supplemented with horse serum (5% final, Invitrogen), antibiotics- Pen/Strep (1% final, Invitrogen), growth factor- EGF (20 ng/mL final, Peprotech, Rocky Hill, NJ), hydrocortisone (0.5 mg/mL final, Sigma), cholera toxin (100 ng/mL final, Sigma), and insulin (10 µg/mL final, Sigma) at 37°C in a humidified atmosphere containing 5% carbon dioxide.

### Experiments

Twenty–four hours before the experiments, MCF-10A cells were seeded at a density of approximately 3×10^5^ cells/well in six-well plates or 5×10^7^/500 cm^2^ square cell culture dishes (Corning). Nicotine was diluted in complete culture medium at the required final serial concentrations. Nicotine dosage experiments were carried out in six well plates for 72 hours and at the end; the number of live cells were calculated using TC10 BioRad® cell counter. These dosage experiments were further analyzed and compared for the nicotine LD_50_ dose (5 mM/811 ng/mL) in 500 cm^2^ plates for 72 hours. After the exposure period, attached cells were harvested for RNA extraction using a Qiagen’s RNeasy® kit following the manufacturer’s protocol. RNA was properly tested for quality on Nano-drop® and Bioanalyzer® before transcriptome sequencing was conducted using Illumina HiSeq®.

### Transcriptome sequencing

RNA libraries were prepared according to the TruSeq® RNA sample preparation guide as per the manufacturer’s recommended protocol (Illumina, San Diego, CA). Total RNA for the three biological replicates from control and nicotine stressed cell populations were transcribed to cDNA. Complimentary DNA samples were then sheared by nebulization (35 psi, 6 min). Duplexes were blunt-ended (large Klenow fragment, T4 polynucleotide kinase and T4 polymerase) and a single 3′adenosine moiety was added using Klenow exo− and dATP. Illumina adapters, containing primer sites for flow cell surface annealing, amplification and sequencing, were ligated onto the repaired ends of the cDNA. Gel electrophoresis was used to select for DNA constructs 200–250 base pairs in size, which were subsequently amplified by 18 cycles of PCR with Phusion polymerase (NEB). These libraries were then denatured with sodium hydroxide and diluted to 3.5 pM in a hybridization buffer for loading onto a single lane of an Illumina HiSeq® flow cell. Cluster formation, primer hybridization and sequencing reactions were according to the manufacturer’s protocol. High throughput sequencing was performed using paired-end, 100 base pair reads. One flow lane was used for cDNA sequencing, yielding an average of 45.26 million reads per sample with mean quality score of >36.3.

### RNASeq data analysis

RNASeq data were analyzed according to the method described by Trapnell et al., [12]. Briefly, TopHat v2.0.3 was used to align the RNAseq reads to the Ensemble GRCh37 genome as provided by Illumina iGenome with annotations. Gene expression was measured for each gene from the Ensembl database by Mapped Fragments per Kilobase of Exon model per Million mapped reads (FPKM) calculated using Cufflinks v2.0.1. We used the program CuffDiff v2.0.1 to test for differential transcript expression and alternative splicing events in each group of cell lines. Default parameters were used for all analyses. Genes were considered differentially expressed after adjusting for multiple testing; p≤0.05. CummeRbund package for R was used to generate scatter plots shown in supplementary data. Genes having evidence of alternative splicing (those with a q-value <0.05 suggesting a change in the relative abundance of different transcripts deriving from a single transcription start site) were identified by CuffDiff. Cufflinks performs transcript inference and abundance estimation followed by differential test of relative abundance using Jensen-Shannon Divergence (JSD) to detect evidence for alternative splicing [13]. In this context, JSD is a measure of change in the relative abundance of multiple transcripts from each locus across two experimental conditions [14]. Gene functions and biological processes were investigated using the PANTHER classification system [15] and transcription factor enrichment was carried out using the Ingenuity Pathway Analysis (IPA) software (http://www.ingenuity.com). Sequence reads are available in NIH Short Read Archive under the experiment accession number SRX254950.

### Quantitative RT-PCR

For each sample, 1 µg of total RNA was reverse transcribed using the Qiagen QuantiTect Reverse Transcription Kit (Valencia, CA) according to the manufacturer’s standard protocol. For Taqman real-time PCR, 1.0 µl of diluted cDNA was used for 20 µl real-time PCR using Taqman Gene Expression Master Mix according to the manufacturer’s standard protocol. Applied Biosystem’s Taqman assays used were as follow: HPCAL4 (Hs04188853_g1), NEAT1 (Hs01008264_s1), Actin (Hs01060665_g1) and 18S rRNA (Hs03928985_g1). Quantitative RT-PCR was carried out in a StepOnePlus real-time PCR system (Applied Biosystems, Foster City, CA).

## Results and Discussion

### Quality control and differential expression analysis

This is the first transcriptome sequencing study of nicotine stressed normal breast epithelial cells. Expression analysis via direct transcriptome sequencing (RNAseq) probes the rarest and most cell- and context-specific transcripts. Furthermore, RNAseq methods are not biased by prior knowledge of splicing and allow the analysis to determine the full repertoire of alternatively spliced isoforms. Finally, RNAseq as a method has been shown to be more quantitative as the number of reads produced from an RNA transcript is a function of that transcript and gene expression rather than a chemical property of probe hybridization that changes with the composition of the sample [16]–[18].

To identify differentially expressed genes and transcripts, we first confirmed the RNAseq data was free of sequencing bias and displayed a uniform coverage across samples (Figure S1). We then normalized the samples (FPKM), calculated a test statistic (p-value) and adjusted p-value (q-value). From this, there were a total of 2015 differentially expressed transcripts (q-value <0.05), 1680 up-regulated and 335 down-regulated (Table S1). A total of 138 transcripts were differentially expressed with ±2 fold change (107 up-regulated and 31 down-regulated) upon nicotine exposure and 39 of these were uncharacterized (Table 1). We used Taqman real-time PCR to validate the top two genes (HPCAL4 and NEAT1) and confirmed their up- and down-regulation respectively (Figure S2).

**Table 1:** List of differentially expressed genes and uncharacterized transcripts (±2 fold change, q<0.05).

Gene	Locus	Fold change	Gene	Locus	Fold change
HPCAL4	1:40144319-40157361	4.71	EMP1	12:13349649-13369708	2.2
TREM1	6:41241842-41254457	4.64	CASP14	19:15160187-15169088	2.2
-	X:39487474-39490677	4.43	UBE2C	20:44441214-44445596	2.19
EFNA2	19:1282818-1378589	3.94	GPNMB	7:23275585-23314730	2.18
-	16:88262665-88265845	3.91	-	4:123715490-123747712	2.18
SPATA22	17:3343307-3461289	3.65	NTSR1	20:61340105-61394123	2.18
KRT85	12:52753789-52761265	3.58	TRNP1	1:27320179-27328107	2.17
FTH1P20	2:181737593-181738141	3.52	IFI27	14:94571181-94583033	2.17
FAM129A	1:184759857-184943746	3.4	C6orf105	6:11711725-11807279	2.17
SPRR2D	1:153012200-153014407	3.31	OAS1	12:113344470-113455556	2.17
GPR56	16:57644563-57698944	3.3	CYP4F3	19:15751693-15773634	2.16
KRT6B	12:52840434-52845971	3.2	TINAGL1	1:32042115-32053288	2.16
IFI44L	1:79085606-79111768	3.2	PHGDH	1:120202420-120286838	2.15
S100A2	1:153532786-153540366	3.19	-	7:106478514-106478920	2.15
MME	3:154741912-154901497	3.15	SYNPO	5:149980641-150038782	2.15
WISP2	20:43285091-43379675	3.12	UNC5B	10:72972326-73062621	2.15
IFI44	1:79115357-79129763	3.11	IGFBP2	2:217497550-217529159	2.14
PROM2	2:95940200-95957056	3.07	WNT7B	22:46316241-46373009	2.14
-	7:151217738-151218277	3.01	-	6:21869148-21869433	2.14
CRCT1	1:152486977-152488486	3	IGSF3	1:117117030-117210375	2.13
SLC22A18AS	11:2907911-2946476	2.9	GSDMA	17:38119225-38134708	2.13
MT1E	16:56659386-56661024	2.89	CGA	6:87790559-87804955	2.12
PTAFR	1:28473676-28520447	2.82	RAET1E	6:150204142-150262882	2.1
-	5:107788477-107790682	2.82	BTBD11	12:107712189-108053421	2.09
SLC7A5	16:87863628-87903094	2.79	-	4:127066284-127069675	2.09
KRT80	12:52562776-52585784	2.77	HEY1	8:80676244-80783994	2.09
CAMP	3:48264836-48266981	2.75	ARC	8:143692409-143695833	2.09
FAT2	5:150883653-150970764	2.74	SH3KBP1	X:19552082-19905719	2.06
HDDC2	6:125589026-125623282	2.7	S100A14	1:153586730-153589970	2.06
STEAP1	7:89511666-89870091	2.69	FAM83F	22:40390952-40440534	2.05
KRT15	17:39669994-39678781	2.64	PLAU	10:75668934-75682535	2.04
SPTLC3	20:12989626-13189886	2.64	-	4:123715490-123747712	2.04
CTSH	15:79213399-79241916	2.63	DHRS3	1:12627938-12677737	2.02
LCE1B	1:152783719-152785585	2.6	SCD	10:102095319-102124640	2.02
HOXB9	17:46684589-46724385	2.59	CLMP	11:122940396-123098985	2.02
CGREF1	2:27309614-27341995	2.58	RAC2	22:37621297-37640488	2.02
-	6:359362-360343	2.58	GM2A	5:150560422-150652294	2.02
SRPX2	X:99899182-99926425	2.58	GPR50	X:150343663-150351320	2.01
-	3:97910175-97910638	2.57	-	22:42770051-42771102	−2.02
KLK5	19:51446559-51456344	2.54	-	1:93520399-93522579	−2.03
SH2D2A	1:156776004-156851642	2.51	-	11:6696821-6699895	−2.03
-	12:132101451-132102489	2.5	-	1:94317575-94319855	−2.04
EVPLL	17:18278850-18292961	2.49	LTF	3:46475832-46526724	−2.06
KRT81	12:52626303-52715182	2.49	SLC25A48	5:135170326-135227452	−2.08
UPP1	7:48127523-48148330	2.45	-	1:160501242-160502784	−2.09
ENTPD3	3:40355292-40494820	2.42	-	8:129998001-130031668	−2.09
-	3:144240244-144244530	2.39	-	11:95039946-95043159	−2.1
MMP2	16:55512741-55540603	2.37	-	1:69041499-69050173	−2.1
ANGPT4	20:853295-896977	2.36	OLFM4	13:53602829-53626196	−2.12
-	14:101759096-101759948	2.36	VRK2	2:58134785-58468588	−2.12
CCDC159	19:11457180-11465681	2.36	-	1:249124073-249126611	−2.13
PAX8	2:113969056-114036527	2.36	-	3:196765047-196766698	−2.13
GRAMD1B	11:123325086-123498482	2.33	WDR96	10:105889296-105992120	−2.14
DDIT4	10:74033677-74035794	2.3	-	14:103524173-103526093	−2.29
KIF21B	1:200938519-200992828	2.29	NEAT1	11:65189789-65213011	−2.3
FAH	15:80444831-80479288	2.29	DEFB129	20:153760-215191	−2.35
LGALS1	22:38071614-38075813	2.28	-	21:34750563-34751650	−2.36
STAC	3:36421835-36589499	2.27	-	6:25644263-25648815	−2.43
-	6:355134-359300	2.27	-	13:91514850-91517039	−2.47
SLCO2A1	3:133651539-133771028	2.27	-	7:112877325-112878981	−2.53
SLC37A2	11:124932962-124960747	2.26	-	6:145278063-145318221	−2.58
PROX1	1:213992977-214214853	2.25	NPAS3	14:33404138-34384243	−2.73
-	X:64992641-65060602	2.25	-	1:69050444-69052682	−2.79
MED24	17:38175349-38210679	2.23	-	18:46521142-46547893	−2.81
VCAN	5:82767283-82878122	2.23	-	X:150472095-150473541	−2.82
CCDC167	6:37450695-37467700	2.22	-	17:80411112-80412283	−3.13
C3AR1	12:7917811-8250367	2.21	-	7:112874561-112876638	−3.23
MLPH	2:238394070-238463961	2.2	-	18:9305458-9307392	−3.52
FLRT2	14:85996487-86095034	2.2	-	5:135053049-135054212	−4.79

Cancer is a multifactorial disease, linked to the number of underlying biological events such as inflammatory response, tumor suppressor genes, oncogenes and transcription factors. An unbiased, all inclusive approach with next-generation sequencing can therefore provide more complete details and help us better understand the missing links. Our results indicated that HPCAL4, TREM1, EFNA2, SPATA22 and KRT85 are the top five upregulated genes and NPAS3, DEFB129, NEAT1, WDR96 and VRK2 are the top five downregulated genes upon nicotine exposure. Hippocalcin like 4 (HPCAL4) and triggering receptor expressed on myeloid cells 1 (TREM1) were highly upregulated genes with greater than four-fold change upon nicotine stress. HPCAL4 is a relatively under-studied gene and has unknown function, but it is reported to be overexpressed in lung carcinoma [19]. The chromosome region 1p34.2, where HPCAL4 is located, is also associated with many types of cancer including breast, lung, neuroblastoma, and colorectal [19]. It is possible that HPCAL4 gene could be the reason for such associations in these cancers. TREM1 is shown to be involved in inflammatory response and is a positive regulator of TNF-α and IL-8 [20]. TREM1 is also a target for matrix metalloproteinases (MMPs) that can cleave extracellular proteins. This transmembrane glycoprotein possesses an Ig-like ectodomain readily shed by MMPs to generate sTREM-1. While membrane-anchored TREM-1 amplifies inflammatory responses, sTREM1 exhibits anti-inflammatory properties [21]. In this study, we have also observed upregulation of MMP2 that may indicate a possible increase in sTREM1 and therefore anti-inflammatory effects. This strengthens previous nicotine findings where it was reported to exhibit immunosuppressive and anti-inflammatory effects [22]. EFNA2 activation promotes the endothelial cell inflammatory response [23], SPATA22 has a role in meiotic DNA repair or recombination and is required for meiotic progress in mouse germ cells [24] and KRT85 is a hair keratin gene that has been reported to be transcriptionally activated by NF-κB effector p65/RelA [25]. The most down-regulated gene, NPAS3, has demonstrated tumor suppressive activity [26] and, therefore, its down regulation in nicotine stressed cells is suggestive of NPAS3’s possible unknown role in increased cancer susceptibility. Interestingly, none of the top regulated genes were detected as differentially expressed in previous microarray studies [9]. This is probably due to several reasons including different cell types used, different dose and exposure times and technological limitations. More striking is the fact that except for TREM1, all other top regulated genes are reasonably new and understudied. This further justifies the reason to sequence transcriptomes for nicotine stressed cells as it probably reveals previously unknown links.

A distinct down-regulated gene is NEAT1, which is a long non-coding RNA (lncRNA) known to be an essential structural determinant of paraspeckles [27]. The functional role of non-coding RNAs are not well defined; however, Chen et. al., have recently proposed a functional role of NEAT1 in the regulation of mRNA export [28]. We are, for the first time, reporting the differential response of lncRNA to nicotine. This creates opportunities to venture into new unexplored avenues in cancer research and exemplifies a greater role for lncRNAs in transcription regulations and cancer development.

### Alternative splicing analysis

Defects in mRNA splicing are an important cause of disease and several studies have reported cancer-specific alternative splicing even in the absence of genomic mutations [29]. We identified 173 genes which showed evidence of alternative splicing (Table S2). Twenty three of those are also differentially expressed (Table S3). It is also important to note that NEAT1 is one of the most down regulated lncRNAs that also presented evidence for alternative splicing upon nicotine stress. The top eighteen genes with square root of the *Jensen-Shannon divergence* >0.8 are shown in Table 2. Disher et. al., suggested that mis-splicing following oxidative stress represents a novel and significant genotoxic outcome and that it is not simply DNA lesions induced by oxidative stress that leads to mis-splicing but changes in the alternative splicing machinery itself [30]. Nicotine is reported to induce oxidative stress in culture cells [31] and therefore, that could be a reason for the detection of aberrant splice variants in nicotine stressed cells. Multiple spliced forms of a single gene could have contrasting biological properties. For example, splice variants of MDM2 displays both oncogenic and growth inhibiting properties [32]. Therefore, to uncover biological significance of alternatively spliced genes upon nicotine stress warrants further investigations.

**Table 2:** Topmost alternatively spliced genes in nicotine stressed cells (sqrt(JS) >0.8).

Gene	Locus	Sqrt(JS)	q_value
ARAP1	11:72396112-72505213	0.833	0.0009
ATG4C	1:62920398-63339980	0.833	0.0009
ATXN2L	16:28833431-28848558	0.833	0.0009
CCDC91	12:28286181-28733149	0.833	0.0009
CGGBP1	3:88101093-88217879	0.833	0.0009
EFNA3	1:155036202-155060014	0.833	0.0009
GNAI2	3:50263723-50296792	0.833	0.0009
GULP1	2:189154435-189460653	0.833	0.0009
IDI2-AS1	10:1034330-1178237	0.833	0.0009
MDH1	2:63348517-64054977	0.833	0.0009
NADKD1	5:36191751-36302379	0.833	0.0009
NIPAL1	4:47849256-48042188	0.833	0.0009
RAB1A	2:65283499-65357422	0.833	0.0009
RASSF8	12:26088948-26232825	0.833	0.0009
RPS11P5	12:133195365-133532892	0.833	0.0009
SH3D19	4:152041394-152246703	0.833	0.0009
WWC3	X:9983601-10112518	0.830	0.0009
ZNF335	20:44561706-44602714	0.825	0.0231

### Gene ontology

**Figure 1:**
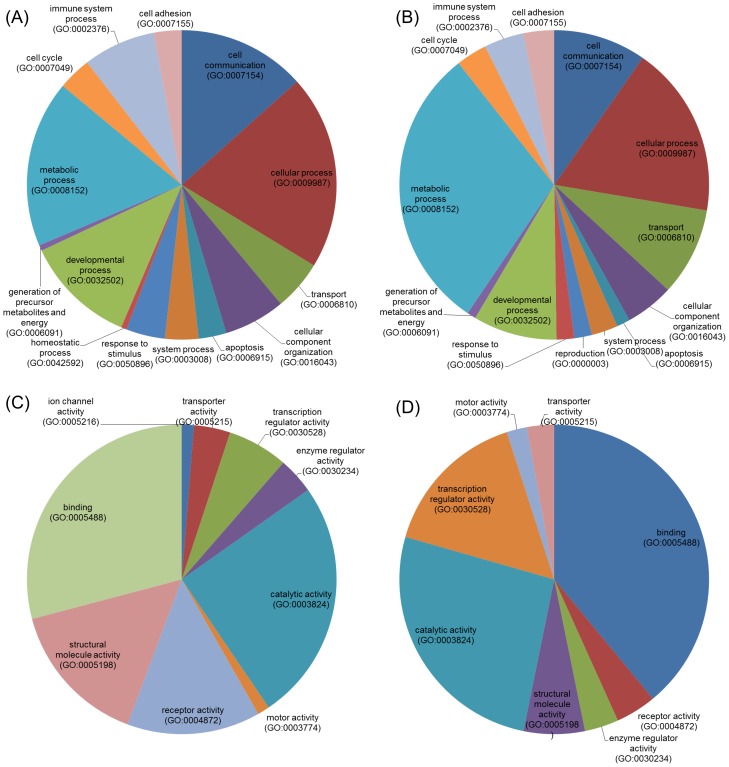
Transcriptome gene ontology (GO) term analysis of differentially expressed and alternatively spliced genes. Pie chart illustrating similarities and differences between GO terms according to the following categories. (1A) Biological process for differentially expressed genes. (1B) Biological process for alternatively spliced genes. (1C) Molecular function for differentially expressed genes. (1D) Molecular function for alternatively spliced genes.

To gain insight into the nature of the genes regulated and alternatively spliced upon nicotine stress, we performed gene ontology analysis using the PANTHER classification system analyzed using binomial test statistics and the Bonferroni correction. Significantly enriched biological processes and gene functions are shown in Figure 1. During nicotine stress, the majority of the genes differentially regulated and alternatively spliced were involved in metabolic, cellular and developmental processes and have catalytic and binding functions. Interestingly, differentially expressed (DE) genes had higher association with structural molecular activity verses the transcriptional regulatory function of alternatively spliced genes. DE genes were further analyzed by Ingenuity Pathway Analysis (IPA) to identify biochemical pathways that may be affected. Figure S3 shows the network of genes that are associated with cancer, the endocrine and nervous systems, which are influenced by nicotine. The NF-κB complex and estrogen receptor are also found in the network of differentially expressed genes suggesting their links to the nicotine stress induced DE genes. It is apparent from this analysis that nicotine affects immune response related genes, indicating the possible influence of nicotine on the modulation of normal immune system activity. Epithelial cancer and dermatological disorders were the top two biological functions associated with nicotine-stressed regulated genes as revealed by IPA. Interestingly, estrogen receptor is linked indirectly to the network suggesting a distant relationship and possible influence of nicotine stress. Also, those genes were associated with cell movement, signalling, proliferation, death and survival functions. Canonical ovarian cancer signalling pathways were significantly associated to these results. Three of the molecules from canonical ovarian cancer pathway, MMP2, CGA and WNT7B were up regulated upon nicotine exposure. Functional significance of alternatively spliced genes evaluated through IPA revealed the top network of genes associated with DNA replication, recombination, and repair (Figure S4).

### Transcription regulator analysis

To identify which transcription regulators regulate the differential expression of those genes described in these experiments, the up- and down regulated genes were reimported into IPA and were analyzed for the enrichment of transcription regulators associated to promoters of differentially expressed genes. There were five up regulator genes (S100A14, HEY1, CGA, IGFBP2 and LGALS1) from the list. Their target molecules, including MMP2 and PROX1, were also up regulated in the study indicating a tight correlation (Table 3). All of the possible transcription regulators whose target genes were differentially expressed are detailed in Table S4. Up regulation of S100A14 is associated with basal-type breast cancers compared to non-basal types [33]. S100A14 is also suggested to be a useful marker for detecting metastatic colorectal and breast cancer [34]. CGA is identified as a new ER-α responsive gene in human breast cancer cells and possibly a strong marker for predicting tamoxifen responsiveness in breast cancer [35]. Significantly higher expression of IGFBP2 is reported in breast cancer tissues compared with benign breast tissue [36]. LGALS1 suppresses T cell-mediated cytotoxic immune responses and promotes tumor angiogenesis [37]. Therefore, over expression of S100A14, CGA, IGFBP2 and LGALS1 in nicotine stressed cells is suggestive of their cumulative role in increased cancer susceptibility in smokers. In our study, nicotine stress up-regulates HEY1 and MMP2. The reverse scenario was observed when evaluating anti-cancer effects of curcumin that resulted into down-regulation of HEY1 and MMP2, due to inactivation of Notch-1 as curcumin inhibited the proliferation and invasion [38]. These suggest the role of nicotine in supporting cancer development and the possibility of curcumin to help reduce the risk and development of cancer in smokers. MMP-2 overexpression has been reported in many neoplasms [39] including ovarian [40] and breast [41] cancers. It has been suggested that MMP-2 may not only be an independent predictor of increased tumor aggressiveness but also important in the activation of other proteases that are directly involved in tumor angiogenesis [42]. It is reported that nicotine increases MMP2 expression and stimulates the esophageal squamous carcinoma cell (TE-13) migration and invasion [43]. Therefore, up-regulation of MMP2 upon nicotine stress in our study further strengthens previous findings and highlights the possibility that MMP2 is a significant biomarker for assessing the risk of cancer in smokers. PROX1 is a transcriptional regulator involved in neurogenesis as well as a variety of cancer types. Alterations in PROX1 are reported in several cancer forms, although it is not always clear whether PROX1 exerts tumor suppressive or oncogenic properties. Colon and brain cancer shows PROX1 over expression, whereas breast cancer reveals reduced expression due to hypermethylation [44]. Therefore, in our study, the effects of elevated PROX1 expression upon nicotine stress are unknown.

**Table 3:** Differentially expressed regulatory genes and their up-regulated targets.

Upstream Regulator	Fold Change	p-value of overlap	Target molecules in dataset
S100A14	2.058	4.81E-03	MMP2
HEY1	2.090	2.97E-03	MMP2,PROX1
CGA	2.117	7.44E-03	CGA,MMP2
IGFBP2	2.143	1.37E-02	IGFBP2,MMP2
LGALS1	2.280	1.16E-02	LGALS1,MMP2

Interestingly, transcription regulator HEY1 is located at cytogenetic band 8q21. Amplification of 8q21 is associated with poor patient prognosis in breast cancer and is independent of MYC [45]. Other nearby interesting genes that were affected by nicotine stress include alternatively spliced NAPRT1 and CPSF1 at cytogenetic band 8q24. Although we did not study the chromosome amplification in this study, the effect of nicotine stress on the transcription regulator HEY1, NAPRT1 and CPSF1 at or near 8q21 is thought provoking. To investigate the overall effects of nicotine on genes per chromosome, we analysed all differentially expressed and alternatively spliced genes (false discovery correction q≤0.05) (Figure S5). Twelve per cent of the genes located at chromosome 19 were differentially expressed and 14 transcripts at chromosome 12 showed evidence for alternative splicing upon nicotine stress. However, chromosome 17 had both a high level of differentially expressed and alternatively spliced transcripts and it is interesting to note that breast cancer marker BRCA1 is also located at chromosome 17. While the reason for chromosomal bias is unknown, expression imbalance is previous documented in hepatocellular carcinoma [46]. Chromosomal bias of differentially expressed transcripts upon nicotine stress therefore warrants future in-depth investigations.

While this pilot study attempts to utilize standardized concentration of nicotine to document physiological and genotoxic effects, lower concentration of nicotine identical to those found in the plasma after smoking may be utilized in the future to further evaluate the effects.

In conclusion, this study reveals that nicotine stress triggers responses from a number of understudied and uncharacterized genes and causes aberrant splicing events that could cumulatively contribute towards the development of cancer and altered immune system activity in smokers. Additionally, genes located at chromosome 12, 17, and 19 show biased sensitivity to nicotine stress indicating unknown biological significance. This implies that nicotine would be an additive in cancer propagation by modulating immune activity and ovarian cancer signalling pathways. This study has wide implications as we present new possible links between genes such as HPCAL4 (up-regulated), NPAS3 (down-regulated) and NEAT1 (alternatively spliced as well as highly down-regulated lncRNA) that could be targeted or monitored to reduce or assess the risk of cancer development in smokers (in particular) and cancer patients. Observations from this transcriptome study thus provide fresh biological insight into nicotine stress on normal breast epithelial cells that can be utilized in clinical settings to assess the harmful effects of nicotine in smokers.

## Supporting Information

Figure S1 **Density graph and scatter plot.** (1A) CummeRbund was used to generate a density graph of the densities of the FPKM values across all genes. The Control and Nicotine samples were very similar, indicating no bias in the sequencing coverage between the samples. (1B) The FPKM values for all genes were plotted for the Control and Nicotine samples, following averaging across replicates and normalization. Each dot represents a gene.
(JPG)

Figure S2 **Validation of top two genes by Taqman real-time RT-PCR.** Taqman real-time RT-PCR validation assessment of HPCAL4 (up regulated) and NEAT1 (down regulated) indicates the general agreement of RNASeq finding and quantitative PCR. Fold changes were relative to the control and normalized to the multiplexed housekeeping genes (Actin and 18S rRNA). Error bars represent standard errors.
(JPG)

Figure S3 **Ingenuity pathway analysis of differentially expressed genes.** Top network from the Ingenuity pathway analysis indicates that DE genes have important role in Cancer. Those shown in red are up-regulated, green are down-regulated and those in white serve to make the indirect connection between the DE genes.
(JPG)

Figure S4 **Ingenuity pathway analysis of alternatively spliced genes.** Top network from the Ingenuity pathway analysis indicates that alternatively spliced genes have association with DNA replication, recombination, and repair.
(JPG)

Figure S5 **Differentially expressed and alternatively spliced transcripts per chromosome and their relative percentages.** (4A) Radar graph indicates relative percentages of DE transcripts per chromosome. (4B) Radar graph demonstrates relative percentages of alternatively spliced transcripts per chromosome. (4C) Table illustrating the total number of differentially expressed and alternatively spliced transcripts per chromosome.
(JPG)

Table S1 **List of all differentially expressed genes and uncharacterized transcripts (q<0.05).**
(XLS)

Table S2 **Transcripts which showed evidence of alternative splicing.**
(XLS)

Table S3 **Overlapping genes that showed differential expression and evidence of alternative splicing.**
(XLS)

Table S4 **IPA transcription factor enrichment analysis presenting all transcription factors that may have influenced differentially regulated genes.**
(XLS)
