# Supplementary figures and images for: Characterizing the Genetic Basis for Nicotine Induced Cancer Development: A Transcriptome Sequencing Study
Source: PLoS One. 2013 Jun 18;8(6):e67252. doi: 10.1371/journal.pone.0067252 (PMC3688980; doi:10.1371/journal.pone.0067252)

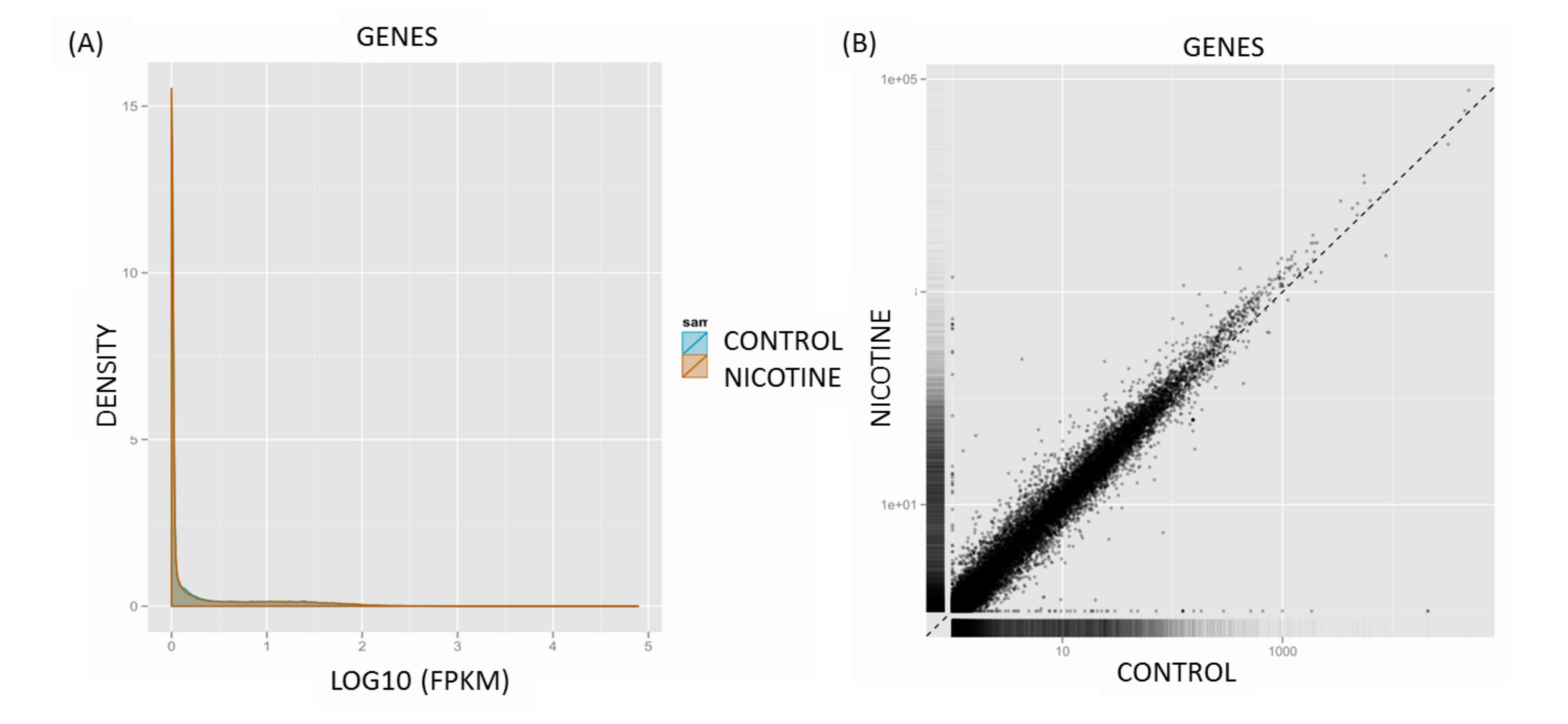

Supplement: Figure S1 — Density graph and scatter plot. (1A) CummeRbund was used to generate a density graph of the densities of the FPKM values across all genes. The Control and Nicotine samples were very similar, indicating no bias in the sequencing coverage between the samples. (1B) The FPKM values for all genes were plotted for the Control and Nicotine samples, following averaging across replicates and normalization. Each dot represents a gene. (JPG) [file pone.0067252.s001.jpg]

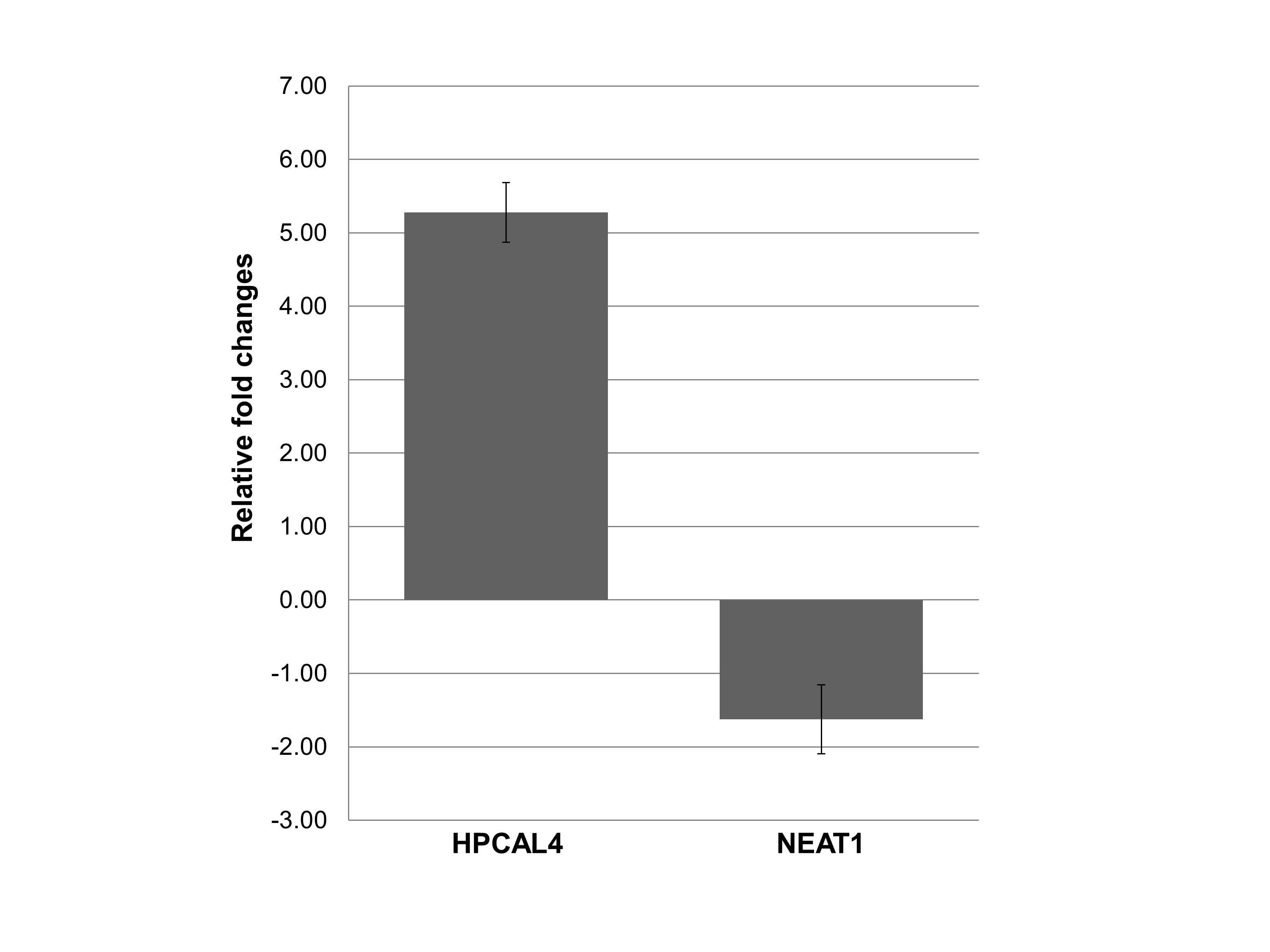

Supplement: Figure S2 — Validation of top two genes by Taqman real-time RT-PCR. Taqman real-time RT-PCR validation assessment of HPCAL4 (up regulated) and NEAT1 (down regulated) indicates the general agreement of RNASeq finding and quantitative PCR. Fold changes were relative to the control and normalized to the multiplexed housekeeping genes (Actin and 18S rRNA). Error bars represent standard errors. (JPG) [file pone.0067252.s002.jpg]

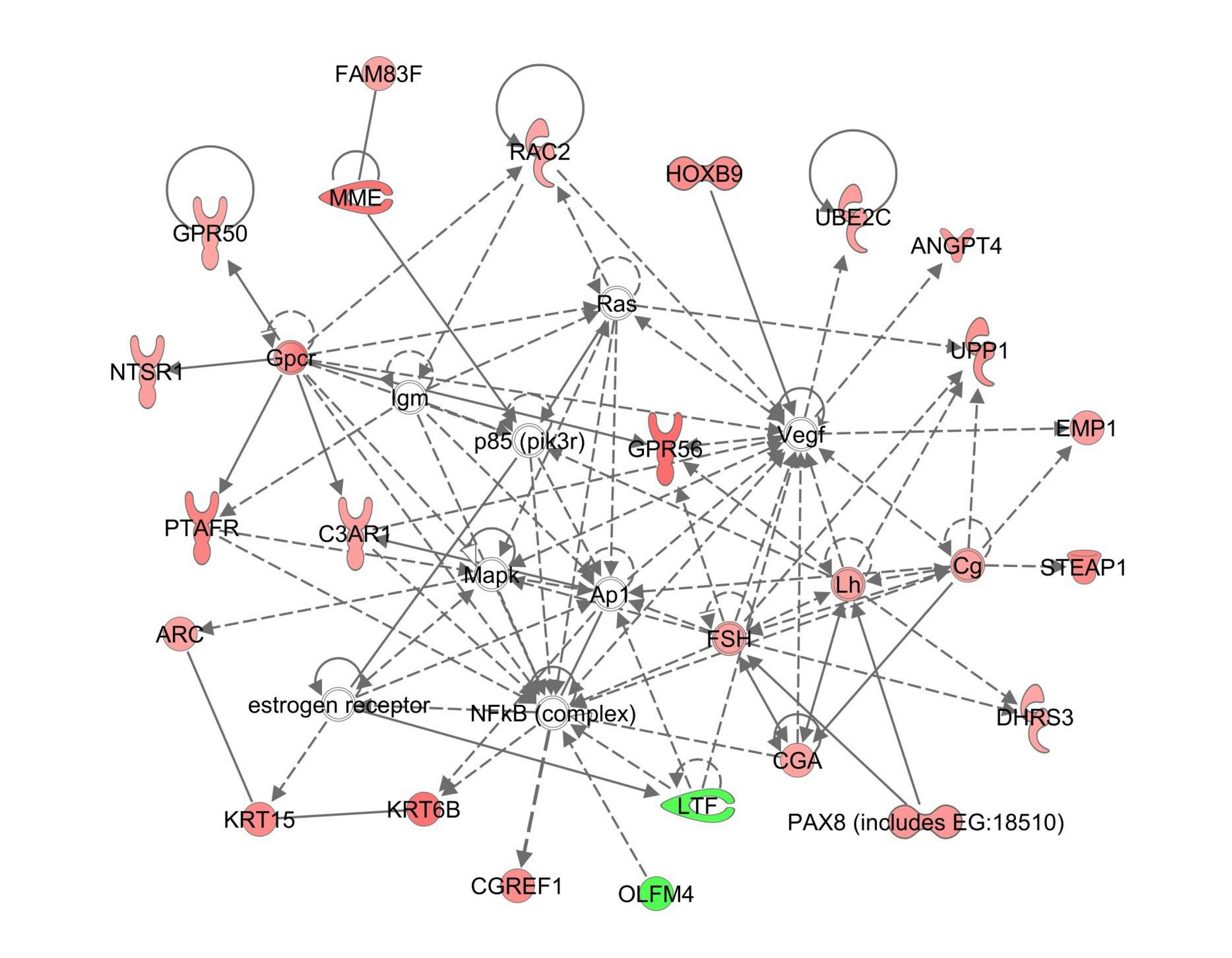

Supplement: Figure S3 — Ingenuity pathway analysis of differentially expressed genes. Top network from the Ingenuity pathway analysis indicates that DE genes have important role in Cancer. Those shown in red are up-regulated, green are down-regulated and those in white serve to make the indirect connection between the DE genes. (JPG) [file pone.0067252.s003.jpg]

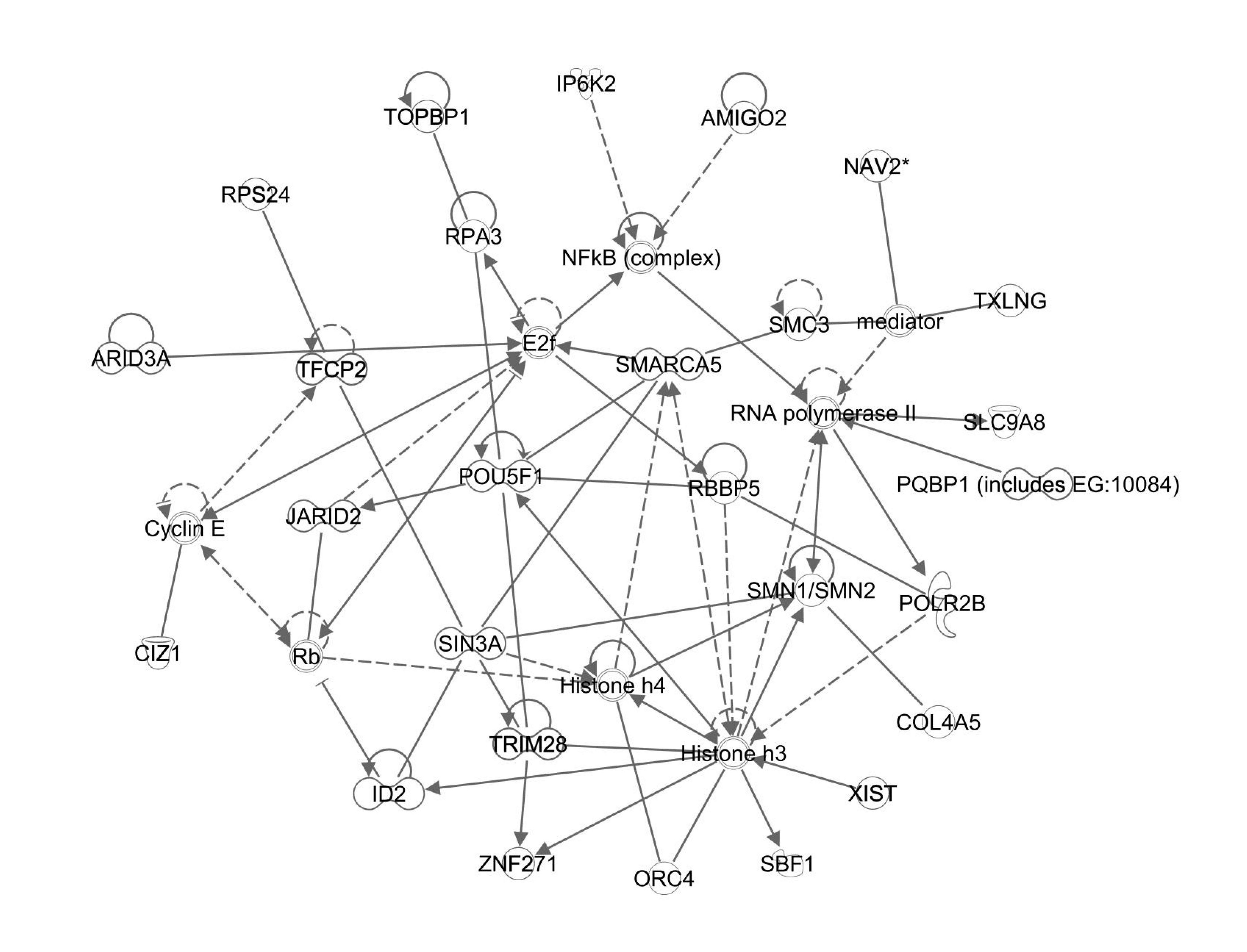

Supplement: Figure S4 — Ingenuity pathway analysis of alternatively spliced genes. Top network from the Ingenuity pathway analysis indicates that alternatively spliced genes have association with DNA replication, recombination, and repair. (JPG) [file pone.0067252.s004.jpg]

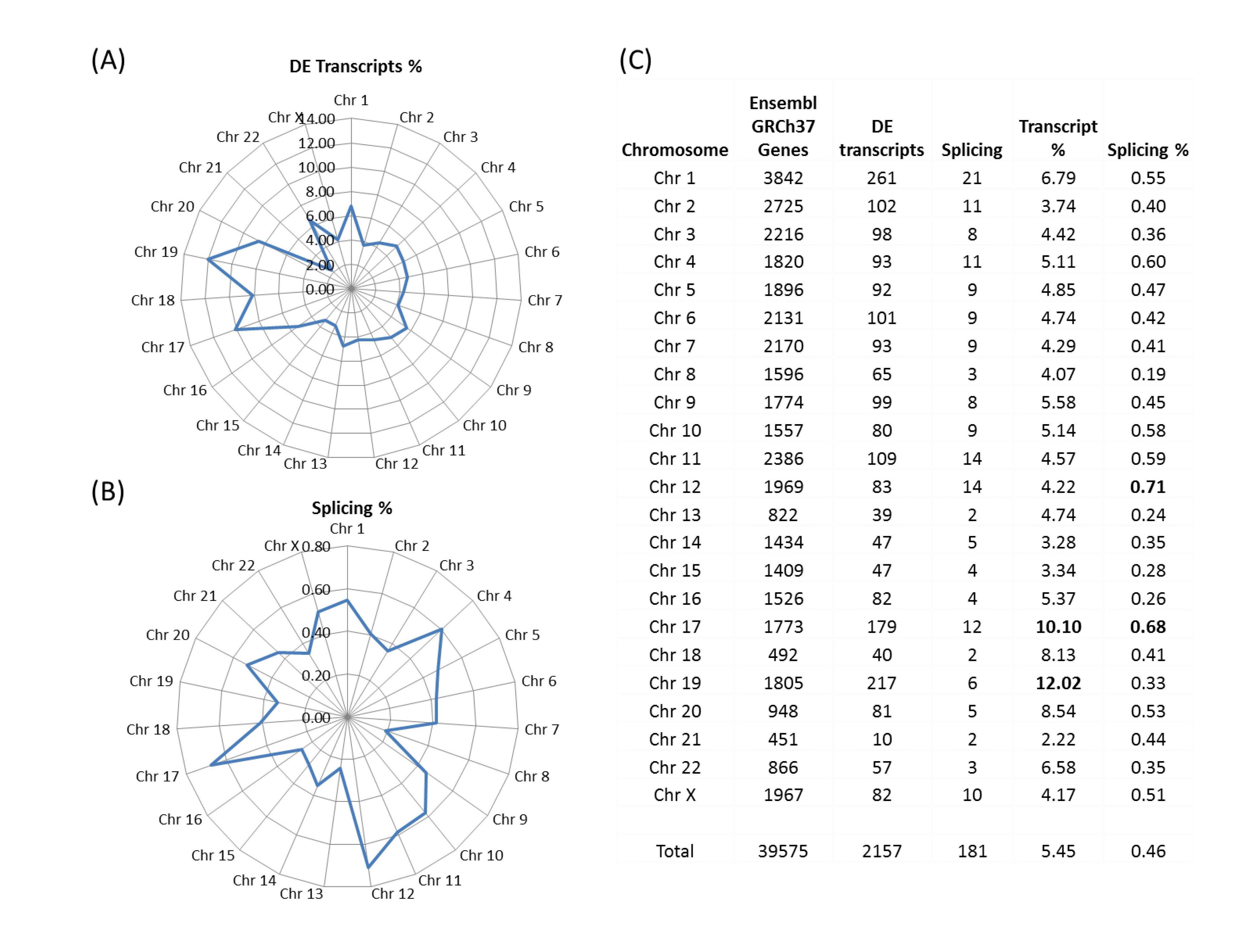

Supplement: Figure S5 — Differentially expressed and alternatively spliced transcripts per chromosome and their relative percentages. (4A) Radar graph indicates relative percentages of DE transcripts per chromosome. (4B) Radar graph demonstrates relative percentages of alternatively spliced transcripts per chromosome. (4C) Table illustrating the total number of differentially expressed and alternatively spliced transcripts per chromosome. (JPG) [file pone.0067252.s005.jpg]
